# Bee honey protects astrocytes against oxidative stress: A preliminary in vitro investigation

**DOI:** 10.1002/npr2.12079

**Published:** 2019-09-17

**Authors:** Amira Mohammed Ali, Hiroshi Kunugi

**Affiliations:** ^1^ Department of Mental Disorder Research National Center of Neurology and Psychiatry National Institute of Neuroscience Tokyo Japan; ^2^ Department of Psychiatric Nursing and Mental Health Faculty of Nursing Alexandria University Alexandria Egypt

**Keywords:** astrocytes, bee honey, neuroinflammation, neuropsychiatric disorders, oxidative stress

## Abstract

**Aim:**

Bee honey is widely used as a bioactive food to improve general health and produce therapeutic benefits in various physical disorders. It also improves cognitive and mood‐related behaviors and symptoms in mice and humans. Still, its direct effect on brain cells is unclear. Here, we examined the effect of whole honey on the survival of astrocytes exposed to oxidative stress.

**Methods:**

Cultured cortical astrocytes were treated with honey (0.1%, 0.3%, 0.5%, 0.6%, 0.7%, 0.8%, 0.9%, 1%, 3%, and 5% [*v*/*v*]) for 24 hours followed by H_2_O_2_ (100 μmol/L) for 3 hours. Cellular viability was assessed with MTT assay.

**Results:**

Honey prevented cellular death in a dose‐dependent manner compared with H_2_O_2‐_treated cells. Honey at 1% concentration had the most significant effect (*P* = .015).

**Conclusion:**

Bee honey exerts a neuroprotective effect through its antioxidant activity.

## Abstract 2

This study tested the effect of bee honey on the survival of astrocytes exposed to H_2_O_2_‐induced oxidative stress. Honey prevented cellular death in a dose‐dependent manner compared with H_2_O_2_‐treated cells. Honey at 1% concentration had the most significant effect (*P* = .015).

## Graphical abstract

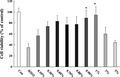

## 1 INTRODUCTION

Honey, a sweet beverage produced by worker honeybees *Apis mellifera*, comprises a rich mixture of various bioactive compounds—phenols, peptides, organic acids, enzymes, minerals, and vitamins. Honey, therefore, demonstrates a range of pharmacological activities (eg, antioxidant, anti‐inflammatory, antilipidemic, antidiabetic, antineoplastic), which encouraged its use to treat several physical disorders.^1^ The high oligosaccharides content of honey contributes to growth of healthy intestinal microflora such *bifidobacteria* (which are used as probiotics that can treat depression), whereas its phenols and internal hydrogen peroxide constituents discourage the growth of harmful pathogens^2^, ^3^ that can cause leaky gut—thinning of intestinal lining that allows passage of bacterial toxins (eg, lipopolysaccharide) into the circulation and then to the central nervous system to induce neuroinflammation, which is evident in depressive disorders.^4^ When honey is orally ingested, it increases levels of vitamin c, trace elements, and antioxidants both in plasma^5^ and in brain. It also enhances the production of neurotrophins such as brain‐derived neurotrophic factor^6^—which is associated with improvement of spatial memory, anxiety and depressive‐like behaviors, and stress response (eg, blood cortisol level) in mice. It is also reported to improve depressive symptoms in the elderly and in postmenopausal women (reviewed in Ref. 7). However, the direct effect of honey on brain cells is not well‐understood.

Astrocytes, which represent a considerable portion of the blood‐brain barrier (BBB), are the main site for the expression of genes contributing to serious disorders such as Alzheimer's disease and Parkinson's disease.^8^ The expression of disease genes such as parkin is associated with activation of astrocytes under severe conditions of oxidative stress, which involves lipid peroxidation and high production of free radicals and mitochondrial reactive oxygen species (mtROS).^9^ In fact, oxidative stress stimulates the initiation of pro‐inflammatory pathways through activation of the NOD‐like receptor protein 3 (NLRP3) inflammasome—a key component that activates cysteine protease caspase‐1, which stimulates the release of the pro‐inflammatory cytokines interleukin‐1β (IL‐1β) and IL‐18.^10^ Both oxidative stress and neuroinflammation induce “reactive astrocytosis”—a dramatic transformation of astrocytes that entails enormous morphological and functional alterations that ruin cellular biomolecules such as DNA and trigger the development of various neuropsychiatric disorders.^11^, ^12^ Given the fact that pharmacological treatments of most psychiatric and neurodegenerative disorders are inadequate in terms of efficacy and adverse effects and that they may even stimulate the production of ROS and accelerate neuronal degeneration,^3^, ^9^ it is worthwhile to examine the neuroprotective effect of natural products that possesses potent antioxidant properties such as bee honey. This study aimed to examine the antioxidant properties of honey in astrocyte cell cultures.

## 2 METHODS

We reseeded astrocytes, isolated from the cortex of 1‐day old pups of Wistar rats (Japan SLC, Inc) according to a previous method,^13^ in a 96‐well microplate at a density of 1.8 × 10^4^ cells/well. Plates containing a medium of complete Dulbecco's Modified Eagle Medium (DMEM) containing 10% fetal bovine serum (FBS)—both purchased from Thermo Fisher Scientific Inc—and 1% penicillin‐streptomycin (Gibco) were kept in an incubator (Thermo Fisher Scientific) at 37°C with humidified air containing 5% CO_2_. After 24 hours, cells were treated with honey (Sugi Garden Co., Ltd.) diluted in sterile distilled water at concentrations of 0.1%, 0.3%, 0.5%, 0.6%, 0.7%, 0.8%, 0.9%, 1%, 3%, and 5% (*v*/*v*) for 24 hours followed by treatment with 100 μmol/L H_2_O_2_ (Fujifilm Wako) for 3 hours. Then, MTT assay (Nacalai Tesque) was performed according to a previous study.^14^ In brief, the medium was replaced with fresh medium containing MTT (0.5 mg/mL) and incubated for 1 hour at 37°C. Then, isopropanol containing 0.04 N HCl was added, and after that a microplate reader was used to measure the optical density at 570 nm. Statistical analysis was performed in SPSS IBM version 22 using one‐way ANOVA with Tukey *post hoc* test. Significance was considered at probability values <.05.

## 3 RESULTS

**Figure 1:**
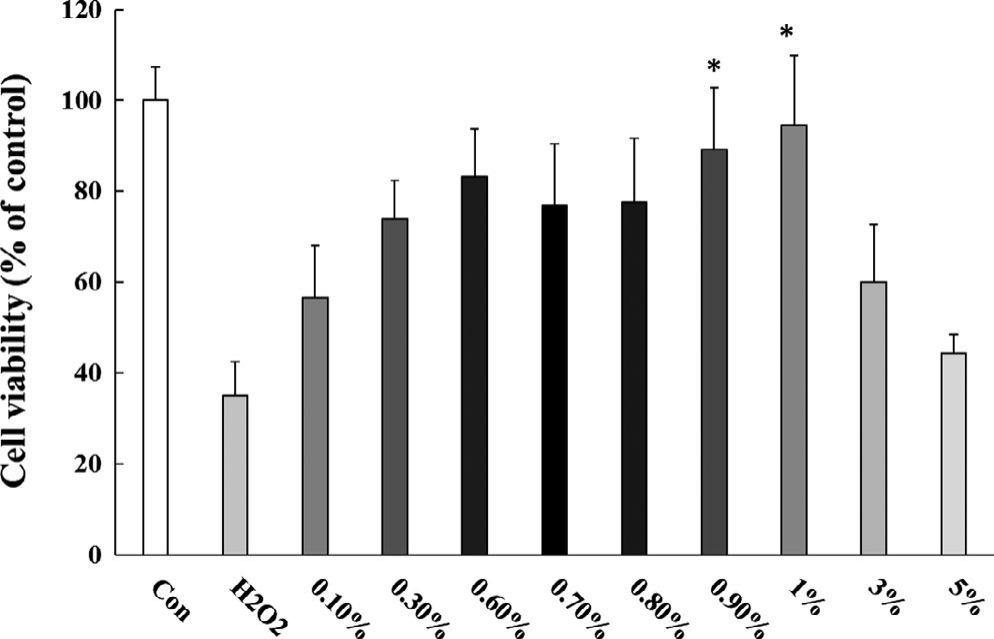
Cell viability after 24‐h treatment of various concentrations of honey to astrocytes exposed to H_2_O_2‐_induced oxidative stress (n = 8). Data represent the mean ± SEM (*P < .05 vs H_2_O_2_ control, one‐way ANOVA followed by Tukey post hoc test)

Treatment with H_2_O_2_ resulted in oxidative stress and considerable cell death compared with untreated control cells (*P* = .005; Figure 1). In order to determine the dose with the highest antioxidant activity, different concentrations of honey were used. Cell viability was remarkably preserved by different concentrations of honey in comparison with H_2_O_2‐_treated cells. Honey at 1% concentration had the most significant effect (*P* = .015), although relatively higher concentrations (3% and 5%) had no significant effect (*P* = .889 and 1.000, respectively). Thus, the effect was observed in an inverted U‐shaped manner.

## 4 DISCUSSION

To our knowledge, this is the first in vitro study that examined the effect of whole honey on astrocytes. The results suggest the neuroprotective effect of honey on cultured astrocytes. Use of whole honey in cell cultures could be a double‐edged sword since its high sugar content may negatively affect cellular osmolarity and cause cell death. Indeed, honey at 1% concentration had the highest antioxidant effect, whereas relatively higher concentrations (3% and 5%) had no significant effect. It is possible that when the amount of internal H_2_O_2_ in honey was small and sublethal, it may elicit adaptive response against subsequent severer oxidative stress.^15^ It has been demonstrated that mild levels of ROS resulted in activation of AMP‐activated protein kinase (AMPK); AMPK decreased internal levels of ROS, which was associated with improved immunity and lengthened life span in *Caenorhabditis elegans*.^16^ In addition, honey is rich in phenols, known to scavenge and remove ROS.^17^ However, when honey was administered at higher concentrations (3% and 5%), detrimental effects may have increased, which may explain the inverted U‐shaped effect. In conclusion, our study, albeit preliminary, provided evidence for protective effect of honey on astrocytes in a dose‐dependent manner, a finding of possible clinical importance. Future studies should examine the effect of honey on the expression of antioxidant genes as well as the related transcription factors.

## 5 LIMITATIONS

The bioactive ingredients of honey could vary based on its botanical origin. Unfortunately, the manufacturer could not confirm the floral origin of honey used in the current study.

## CONFLICT OF INTEREST

The authors declare no conflict of interest.

## AUTHOR CONTRIBUTION

HK contributed to the hypothesis development, overall design of the research, cowrote, and revised the manuscript. AMA performed the laboratory experiment, analyzed data, and wrote the first draft of the manuscript. All authors read and approved this paper.

## ANIMAL STUDIES

All experiments were performed in accordance with the Guidelines for Care of Laboratory Animals of the National Center of Neurology and Psychiatry, Tokyo, Japan.

## Data Availability

Raw data of the current experiment are included in Supporting Information.
